# Asymmetric 3-premolar extraction for an adult class II division 2 subdivision with deep bite and midline deviation: A case report

**DOI:** 10.1097/MD.0000000000047004

**Published:** 2026-01-02

**Authors:** Viet Anh Nguyen, Thi Bao Yen Ngo

**Affiliations:** a Faculty of Dentistry, Phenikaa University, Hanoi, Vietnam; b Private Practice, Viet Anh Orthodontic Clinic, Hanoi, Vietnam.

**Keywords:** adult orthodontics, camouflage orthodontics, esthetic appliances, lingual orthodontics, torque control

## Abstract

**Rationale:**

Class II subdivision with division-2 features and deep bite presents concurrent sagittal, transverse, and vertical challenges. Asymmetric extraction may correct the midline and crowding while enabling incisor decompensation and esthetic improvement with limited patient compliance.

**Patient concerns:**

A 39-year-old woman sought reduction of “protrusion” and improvement of smile esthetics, along with alignment of crowded maxillary incisors and correction of a rightward mandibular midline deviation.

**Diagnoses:**

Skeletal class II pattern with a right side class II subdivision, division-2 incisor inclination, deep bite, increased overjet, moderate crowding, and mandibular midline deviated 3 mm to the right.

**Interventions:**

An asymmetric 3-premolar extraction protocol (both maxillary first premolars and the mandibular second premolar on the class II side) was combined with controlled incisor mechanics, careful vertical control for bite leveling, space closure, and asymmetric finishing goals.

**Outcomes:**

Treatment achieved full alignment, coincidence of dental and facial midlines, normalization of overjet, overbite, and improvement of smile esthetics and lip posture. The left side finished in class I, and the right side in a deliberate functional class II molar relationship consistent with the asymmetric plan. Radiographic skeletal and dentoalveolar measurements indicated a clinically meaningful reduction in the sagittal discrepancy.

**Lessons:**

For selected adult class II subdivision cases with division-2 features and deep bite, asymmetric 3-premolar extraction offers a predictable, compliance-light alternative to 4-premolar extraction or distalization. Intentional functional class II finishing on the affected side can be compatible with stable occlusion when midlines, overjet, overbite, and incisor torque are properly managed.

## 1. Introduction

Class II malocclusion is among the most prevalent sagittal discrepancies, with an estimated global prevalence of about 20% in the permanent dentition; deep overbite is also frequent, and class II division 2, although less common, is typically associated with retroclined maxillary incisors and a deepbite pattern.^[1,2]^ Within this spectrum, class II subdivision – defined by a unilateral class II relationship and midline deviation toward the class II side – is common and often reflects underlying dentoalveolar and skeletal asymmetries. Contemporary cone beam computed tomography-based studies suggest that subdivision patterns may account for up to half of class II cases and have a reported population prevalence of 12% to 37%, underscoring their clinical relevance and the need for 3-dimensional assessment.^[3–5]^

In adults with class II division 2 features and deep bite, diagnosis should integrate facial symmetry and midline analysis, occlusal evaluation, and cephalometric measurements, supplemented by cone beam computed tomography when clarification of the center of asymmetry or transverse discrepancies is required. ^[4,5]^ Management options include symmetric extractions (4 premolars), asymmetric extraction protocols (e.g., 2 maxillary first premolars plus a mandibular premolar on the class II side), non-extraction distalization with skeletal anchorage, asymmetric functional appliances, clear aligners with elastics, and orthognathic surgery when skeletal disharmony predominates.^[6–8]^ Comparative evidence indicates that 3-premolar asymmetric extraction can provide efficient midline correction with long-term stability comparable to 4-premolar protocols, while reducing reliance on heavy class II mechanics.^[9]^ Deepbite correction in this context demands careful vertical control to preserve incisor torque and soft tissue balance.^[10]^

Adjunctive soft tissue procedures, such as diode-laser gingivoplasty or crown lengthening, can further refine smile esthetics when excessive gingival display or altered passive eruption contributes to the chief complaint.^[11]^ Nevertheless, there is limited evidence addressing adult class II division 2 subdivision with deep bite and midline deviation managed with an asymmetric 3-premolar extraction protocol (2 maxillary first premolars plus a unilateral mandibular second premolar), particularly when skeletal and soft tissue changes are quantified, and the affected side is intentionally finished in a functional class II molar relationship.^[9,12]^

This case report describes the diagnosis and management of an adult class II division 2 subdivision (right) with deep bite and mandibular midline deviation treated with asymmetric 3-premolar extraction. The treatment aimed to resolve crowding, correct the mandibular midline, and normalize overjet and overbite; retract and decompensate incisors to improve smile esthetics and lip posture; and contextualize the biomechanical choices among contemporary alternatives.

## 2. Case presentation

### 2.1. Diagnosis and etiology

A 39-year-old woman presented seeking reduction of mouth “protrusion” and improvement of smile esthetics, alongside relief of maxillary anterior crowding. Medical and dental histories were noncontributory. Extraorally, the patient exhibited a relatively symmetrical and harmonious face with well-balanced facial thirds. The profile was mildly convex with a low smile line, exposing about 30% of the lower anterior teeth, and the buccal corridors were within normal limits. The mandible was deviated approximately 3 mm to the right.

**Figure 1.**
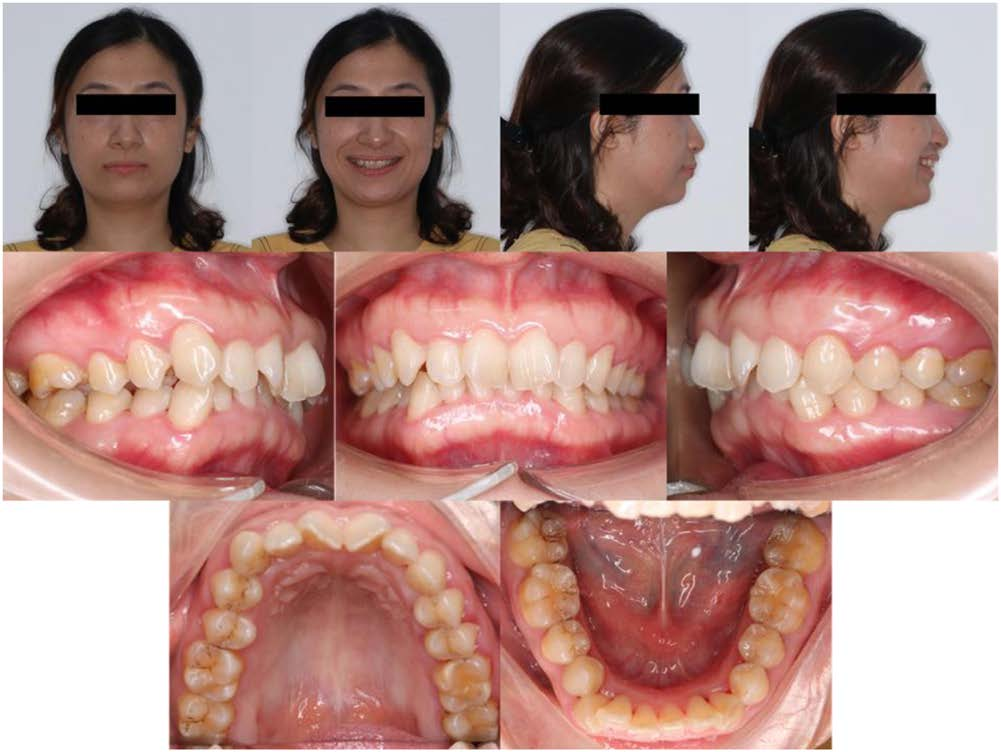
Pretreatment extraoral and intraoral photographs.

Intraorally, there was bimaxillary anterior irregularity with retroclined maxillary central incisors, a unilateral right full-cusp class II buccal-segment relationship, and a class I relationship on the left side (Fig. 1). The mandibular dental midline was deviated 3 mm to the right, with increased overjet (5.12 mm) and a deep overbite (5.44 mm; 80% coverage of the mandibular incisors). The mandibular curve of Spee was accentuated. There was moderate crowding in both arches, with a space deficiency of 7.8 mm in the maxilla and 6.2 mm in the mandible, and the lower right canine was erupted outside the arch.

**Figure 2.**
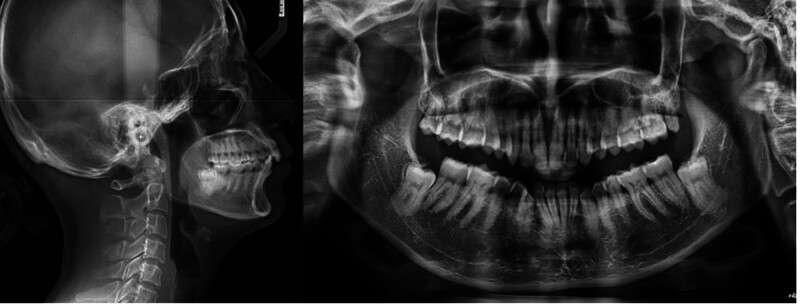
Pretreatment lateral cephalogram and panoramic radiograph.

Cephalometric analysis revealed a skeletal class II pattern, characterized by a relatively normal maxilla (sella-nasion-A point 80.82°) but a retrusive mandible (sella-nasion-B point 73.49°), resulting in an point A-nasion-point B (ANB) angle of 7.32° and a Wits appraisal of +8.88 mm (Table 1). The Frankfort mandibular plane angle was 27.19°, indicating a normodivergent vertical pattern. Dentally, the maxillary incisors were retroclined (U1-sella-nasion [U1-SN] 91.03°, U1-nasion-point A [U1-NA] 13.22°, 2.13 mm), whereas the mandibular incisors were proclined and protrusive (incisor mandibular plane angle 100.55, L1-nasion-point B [L1-NB] 28.27°, 7.79 mm). Soft tissue analysis showed near-balanced lips relative to the E-line (upper lip + 0.12 mm, lower lip + 0.99 mm), but with an acute nasolabial angle of 87.1°. Panoramic radiographic evaluation revealed asymmetry in the lower jaw, normal crestal bone level, and 2 impacted lower wisdom teeth (Fig. 2).

**Table 1:** Measurements obtained from pretreatment and posttreatment lateral cephalometric radiographs.

Measurements	Pretreatment	Posttreatment	Norm
Skeletal			
SNA (°)	80.82	79.82	81.1 ± 3.7
SNB (°)	73.49	72.84	79.2 ± 3.8
ANB (°)	7.32	6.98	2.5 ± 1.8
FMA (°)	27.19	28.20	25.0 ± 4.0
Wits appraisal (mm)	8.88	4.57	0.4 ± 2.3
Dental			
Upper incisor/SN (°)	94.03	86.85	105.3 ± 6.6
Upper incisor/NA (°)	13.22	7.03	22.0 ± 5.0
Upper incisor/NA (mm)	2.13	0.70	4.0 ± 3.0
L1-MP (°)	100.22	100.84	90.0 ± 3.5
Lower incisor/NB (°)	28.27	29.27	25.0 ± 5.0
Lower incisor/NB (mm)	7.79	7.64	4.0 ± 2.0
Interincisal angle (°)	131.19	136.13	128.0 ± 5.3
Overjet (mm)	5.12	3.61	2.0 ± 2.0
Overbite (mm)	5.44	3.92	2.0 ± 2.0
Soft tissue			
Upper lip/E-line (mm)	0.12	-0.32	0.0 ± 2.0
Lower lip/E-line (mm)	0.99	0.31	0.0 ± 2.0
Nasolabial angle	87.1	101.1	105.0 ± 10.0

ANB = point A-nasion-point B, FMA = Frankfort mandibular angle, L1-MP = lower incisor to mandibular plane, NA = nasion-point A, NB = nasion-point B, SN = sella-nasion, SNA = sella-nasion-point A, SNB = sella-nasion-point B.

Based on these findings, the diagnosis was unilateral right class II malocclusion on a skeletal class II base with a class II division 2 incisor pattern, deep overbite, increased overjet, moderate bimaxillary anterior crowding, and right deviation of the mandibular midline.

### 2.2. Treatment objectives

The treatment objectives were to relieve the maxillary and mandibular anterior crowding, including alignment of the displaced mandibular right canine, and to correct the mandibular dental midline deviation toward coincidence with the facial midline. Additional goals were to normalize the overjet and overbite with appropriate vertical control to prevent bite deepening, and to retract and decompensate the incisors to enhance smile esthetics, lip posture, and overall facial balance. Finally, the treatment aimed to establish a functional and stable occlusion with bilateral class I canine relationships and acceptable molar intercuspation.

### 2.3. Treatment alternatives

Multiple treatment alternatives were carefully evaluated and discussed with the patient, each presenting distinct biomechanical implications, advantages, and limitations.

The first approach involved the combined extraction of all second premolars, comprehensive orthodontics, and orthognathic surgery. Extraction of bilateral maxillary and mandibular second premolars to decompensate dentoalveolar compensations, full orthodontic leveling and alignment, followed by corrective orthognathic surgery to normalize skeletal relationships and optimize facial profile. The advantages of this option were definitive correction of the skeletal discrepancy and the highest probability of obtaining ideal dental and skeletal relationships (class I molar and canine relationships, optimal overjet and overbite, and improved facial profile and midline coordination). However, surgical morbidity and associated perioperative risks in a middle-aged patient, increased total treatment time and cost, and greater patient burden may be unacceptable to patients who decline surgery.

The second treatment alternative involved the extraction of the maxillary second premolars only as a nonsurgical camouflage approach. Both maxillary second premolars would be removed to create space for retraction of the maxillary incisors and reduction of the overjet, whereas mandibular crowding and leveling would be managed without extractions, using anchorage reinforcement (temporary anchorage devices [TADs] or auxiliaries) as required. The key to this strategy was avoiding orthognathic surgery and its risks, which may partially reduce overjet and improve dental esthetics with less invasiveness. But this does not correct the underlying skeletal discrepancy; limited improvement of the facial profile. Mandibular Spee curve and crowding are unlikely to be adequately resolved with maxillary extractions alone, and there is substantial anchorage demand with an increased risk of anchorage loss and inadequate midline correction.

The third option proposed a symmetric 4-premolar extraction pattern combined with nonsurgical camouflage. Extraction of maxillary first premolars and mandibular second premolars to provide symmetric space for alignment and leveling while attempting to camouflage the sagittal discrepancy. This approach provided space to level the mandibular arch without excessive proclination of lower incisors and facilitates reposition of the lower right canine. However, given the marked skeletal discrepancy, camouflaging may leave residual class II tendency and excessive overjet; anchorage demands remain high, and profile improvement will be less than surgical correction.

The last and selected treatment strategy involved asymmetric extraction, including maxillary first premolars and a mandibular second premolar, in combination with orthodontic camouflage. Extraction of both maxillary first premolars and the mandibular left second premolar aimed to create space for retraction of maxillary incisors, allow mandibular leveling, and enable translation of the mandibular midline to the left to correct the 3-mm right deviation. Reinforced anchorage (TADs) and controlled biomechanics will be employed. This approach provided adequate space to retract maxillary incisors and improve overjet and upper-lip support without requiring orthognathic surgery. The unilateral mandibular extraction permits controlled leveling of the curve of Spee and targeted midline correction while addressing the buccally displaced lower right canine. Additionally, this option was less invasive than surgery, with a lower cost and faster recovery. However, asymmetric extraction mechanics are complex and demand strict anchorage control to avoid undesired tipping, occlusal cant, or residual asymmetry. Camouflage does not alter the skeletal base; facial profile improvement will be limited relative to surgical correction. Precise finishing is required to avoid residual occlusal or midline discrepancies.

### 2.4. Treatment progress

Orthodontic treatment was initiated with the placement of lingual metal brackets (ADB, Medico, Gyeonggi-do, Korea) on the maxillary and mandibular arches. Leveling and alignment commenced using a 0.012-inch nickel–titanium (NiTi) archwire, followed by progression to 0.014-inch, 0.016-inch NiTi archwires for both arches, then 0.016 × 0.022-inch NiTi archwire for the maxillary arch and 0.016 × 0.016-inch NiTi archwire for the mandibular arch. This initial phase aimed to relieve crowding and align both arches.

**Figure 3.**
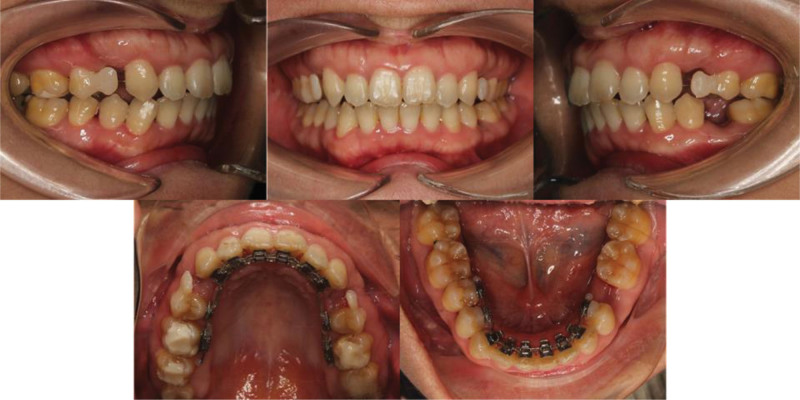
Progress records after premolar extractions with temporary replacements.

Two months following initial bonding, the maxillary first premolars and the mandibular left second premolar were extracted under local anesthesia. Then the 0.016 × 0.022-inch stainless steel (SS) archwire was replaced for the maxillary arch for space closure. Meanwhile, the 0.012-inch NiTi was replaced for the mandibular, and temporary teeth in the position of the extracted teeth were made according to the patient’s wishes (Fig. 3).

The elastic chains were continuously replaced in the upper jaw, while the lower jaw was sequentially leveled with 0.014-inch and 0.016-inch NiTi archwires, followed by 0.016 × 0.016-inch and 0.016 × 0.022-inch SS archwires for re-leveling. After 6 months of space closure, the maxillary archwire was replaced with a 0.017 × 0.025-inch SS archwire to provide torque control of the maxillary incisors. One month later, labial brackets were bonded to the mandibular left first and second molars, and elastic chains extending from the mandibular left first molar to the maxillary left second incisor were applied to facilitate class II elastic mechanics on the left side.

At 23 months into treatment, space closure was completed; however, a slightly increased overjet and residual class II relationship persisted. Power hooks were added between the maxillary central and lateral incisors, and 2 TADs (diameter 1.6 mm, length 10 mm) were placed between the maxillary molars to correct the remaining anteroposterior discrepancy. Each TAD was loaded with approximately 150 g of force per side via elastomeric chains attached to power hooks between the maxillary central and lateral incisors on a 0.017 × 0.025-inch SS archwire. The line of action of this force was planned to pass close to the center of resistance of the maxillary incisors, thereby promoting bodily retraction with torque control while reinforcing posterior anchorage. During the finishing stage, left-sided class II elastics, cross elastics, and vertical triangular elastics were employed to improve the midline, inter-arch coordination, and molar intercuspation.

After 26 months of active treatment, all brackets were removed. Fixed lingual retainers were bonded to both the maxillary and mandibular arches. Residual adhesive was carefully removed using silicone polishing tips to preserve enamel integrity. The patient was transitioned to the retention phase with clear overlay retainers and scheduled for follow-up appointments every 6 months to monitor long-term stability and detect potential relapse.

### 2.5. Treatment results

The treatment achieved the objectives of alignment of both arches, correction of the mandibular midline, improvement of overjet, overbite, and enhancement of smile esthetics. Extraorally, the skeletal deviation of the mandible to the right was still evident; however, dental alignment and arch coordination produced a more harmonious smile. The patient’s facial profile was also improved owing to the reduction in upper lip protrusion, resulting in a more balanced soft tissue appearance.

**Figure 4.**
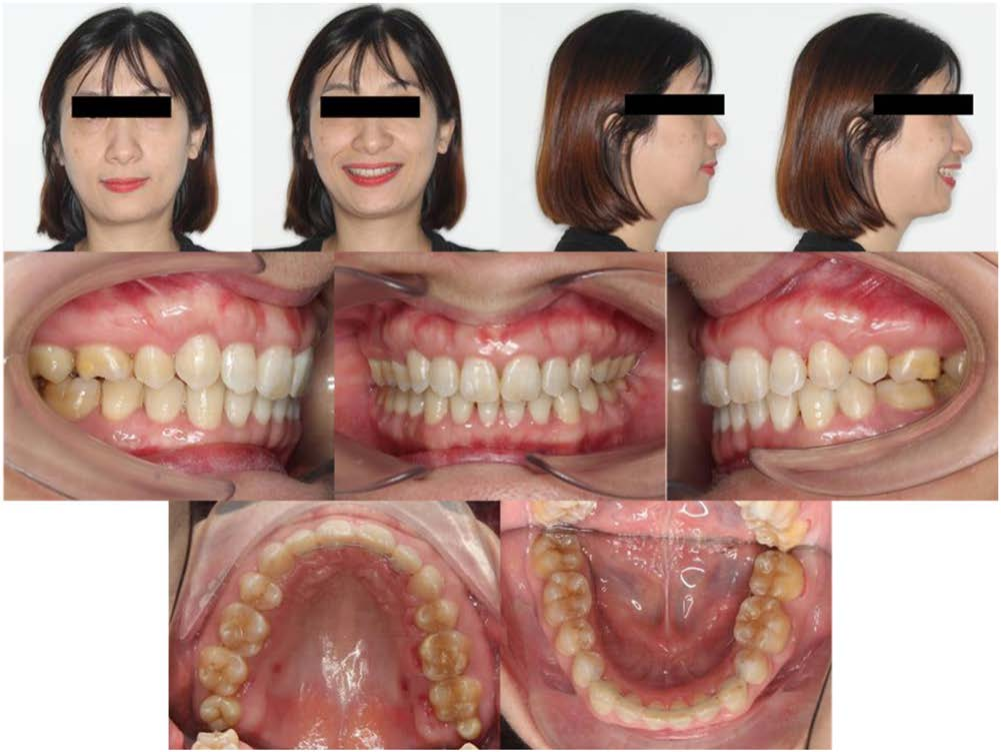
Posttreatment extraoral and intraoral photographs.

In dental alignment, leveling and midline correction were successfully accomplished. The maxillary and mandibular dental midlines were coincident with the facial midline. The previously buccally displaced mandibular right canine was successfully aligned into the arch. The canines finished in a bilateral class I relationship, while the left first molar was class I, and the right first molar remained full-cusp class II with good interdigitation (Fig. 4). Incisor retraction is reflected by the reduction in U1-NA (from 13.22° and 2.13 mm to 7.03° and 0.70 mm), with almost maintenance of lower incisor angulation (L1-NB). Overjet and overbite were improved to functional values (OJ 3.61 mm; OB 3.92 mm). The Wits appraisal improved substantially (from +8.88 mm to +4.57 mm), suggesting a clinically meaningful reduction in the dentoalveolar sagittal discrepancy despite a small change in ANB.

**Figure 5.**
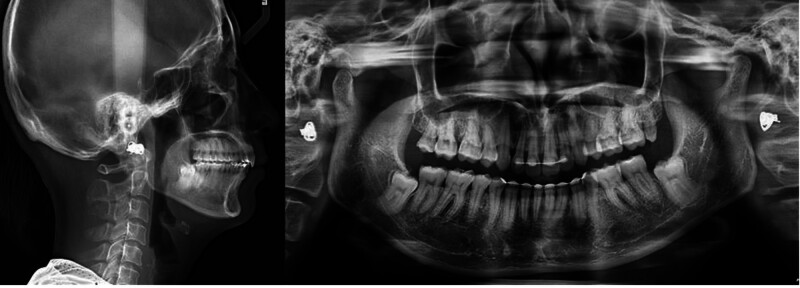
Posttreatment lateral cephalogram and panoramic radiograph.

**Figure 6.**
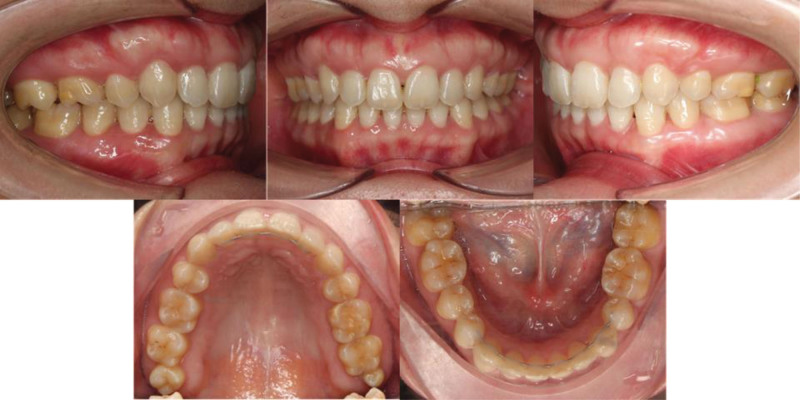
Two-year post-retention extraoral and intraoral photographs.

Soft tissue measurements show slight posterior positioning of the upper lip relative to the E-line and minor reduction in lower lip prominence, with improvement in the nasolabial angle. No adverse periodontal complications or clinically significant root resorption were noted on routine radiographic follow-up. The panoramic x-ray showed no significant changes in the bone levels compared to pretreatment (Fig. 5). Two-year post-retention records confirmed a stable occlusion with no reopening of extraction spaces (Fig. 6).

## 3. Discussion

The treatment outcomes in this case achieved initial objectives, with optimal alignment of both arches, correction of the mandibular midline deviation, and improved overjet, overbite, and smile esthetics. Employing an asymmetric extraction strategy addresses the class II subdivision discrepancy while maintaining a balanced occlusion and facial harmony. Notably, the right (class II side) occlusion was finished in a functional class II molar relationship with a class I canine, whereas the left side achieved a full class I occlusion – all with coincident dental midlines. This outcome is consistent with reports that asymmetric 3-premolar extraction can successfully correct midline deviations and resolve unilateral class II relationships without compromising overall occlusion or aesthetics.^[12,13]^ An added benefit of our chosen plan was reduced reliance on patient compliance; unlike the conventional 4-premolar extraction approach (which typically requires extensive class II elastic wear to correct the asymmetric molar relationship), the 3-premolar protocol allowed us to achieve our goals with minimal extraoral force usage. Furthermore, extracting and retracting the upper premolars provided space to retroposition the protrusive incisors, resulting in a more posterior upper lip position and improved profile. Importantly, treatment was completed with no notable root resorption or periodontal issues, indicating that our mechanics were biologically sound and that the occlusion achieved is likely to be stable.

In planning this case, we considered several alternative approaches. A traditional option for class II subdivision malocclusion is a symmetric extraction of 4 premolars (2 upper and 2 lower) to aim for bilateral class I molar relationships. However, as noted above, achieving a class I result on the initially class II side with 4 extractions often requires prolonged patient cooperation with class II elastics or other inter-arch mechanics, while also sacrificing an additional tooth unnecessarily.^[13]^ Such compliance-dependent strategies can lengthen treatment time and introduce variability in outcomes. Another potential approach was a non-extraction correction using differential distalization or functional appliances.^[14,15]^ For instance, one could attempt to distalize the upper right segment with appliances (e.g., buccal miniscrew anchorage or a unilateral distalizer) or use intermaxillary elastics to correct the molar relationship. While feasible, these methods again rely on patient compliance and may yield less predictable midline correction in an adult, especially given the skeletal class II base. Growth modification was not an option here (the patient was no longer growing), but it’s worth noting that functional appliances have been used asymmetrically in class II subdivision cases to stimulate mandibular advancement on the deficient side.^[16]^ A recent case report by Lorenzoni et al illustrated that an asymmetrically installed Herbst appliance, followed by fixed appliances, successfully corrected a unilateral class II molar relationship and midline deviation, with improved lip posture and facial profile; notably, the results remained stable 2 years posttreatment.^[17]^ While this non-extraction approach can be effective, it introduces greater biomechanical complexity and patient burden (e.g., wearing a fixed functional appliance) compared to extractions.

The asymmetric 3-premolar strategy concentrated space creation on the class II side, allowing midline correction with reduced reliance on heavy inter-arch mechanics. Quantitatively, overjet decreased from 5.12 mm to 3.61 mm and overbite from 5.44 mm to 3.92 mm, with Wits 8.88 mm to 4.57 mm and minimal ANB change (7.32°–6.98°), indicating primarily dentoalveolar compensation. Maxillary incisor torque control (U1-SN 94.03°–86.85°) coincided with an improved nasolabial angle (87.1°–101.1°), supporting the soft tissue benefit of controlled anterior retraction.

In comparison with recent literature, randomized or comparative evidence suggests that 3-premolar protocols in type-1 class II subdivision achieve at least comparable efficiency to 4-premolar extraction and tend to yield better midline outcomes and occlusal success, with less dependence on prolonged class II elastics.^[9]^ Non-extraction options – unilateral distalization with TADs or asymmetric functional appliances (Herbst and Forsus) – remain viable but introduce additional compliance and biomechanical demands.^[7]^ Our results align with these trends: targeted space on the affected side delivered midline correction and acceptable finishing without adverse periodontal or root outcomes.

Yet another emerging alternative is minimal extraction therapy. Pothuri et al reported treating a class II division 2 subdivision malocclusion with the extraction of a single premolar as a “new trend” approach.^[18]^ In that case, removing just 1 premolar (on the appropriate side) was sufficient to correct the asymmetry, allowing the midlines to coincide and achieving class I canine relationships bilaterally. This underscores that in select mild subdivision cases, 1 well-chosen extraction can resolve the discrepancy.^[19]^ In our patient’s case, however, a single-tooth extraction would likely not have generated enough space to resolve the significant crowding, overjet, and deep bite. We therefore proceeded with the 3-premolar extraction strategy as the most balanced solution – it provided ample space for incisor retraction and bite leveling, corrected the midline discrepancy directly, and minimized the need for patient-dependent mechanics. Additionally, Harding et al described a recent class II case in which mesialization of the second and third molars was used to replace a missing first molar, further illustrating how carefully planned, minimal extraction mechanics can individualize space management in selected class II patients.^[20]^ Orthognathic surgery (mandibular advancement) was also considered, given the skeletal class II relationship, but the successful dental compensation achieved here shows that a nonsurgical approach was sufficient and avoided the risks and costs associated with surgery.^[21]^

In addition to the intended sagittal and transverse corrections, particular attention was paid to potential side effects commonly associated with asymmetric extraction protocols and unilateral mechanics, including occlusal plane canting, midline instability, and occlusal plane cant.^[22,23]^ Recent reviews have emphasized that off-center force systems with TADs and prolonged use of inter-arch elastics may predispose to occlusal plane tilting and unwanted midline shifts in asymmetric cases, particularly in class II subdivision malocclusions.^[24,25]^ In the present patient, these risks were minimized by coordinating the archwires symmetrically, working with rectangular SS wires, carefully controlling the magnitude and direction of forces applied to the TADs, and limiting the duration and intensity of unilateral class II elastics. Posttreatment frontal photographs and cephalometric superimpositions demonstrated no clinically relevant occlusal plane cant, stable maxillary and mandibular dental midlines coincident with the facial midline, and a balanced transverse smile display.

This is a single-patient case report; therefore, external validity is limited, and causal inferences cannot be drawn. Long-term follow-up beyond post-retention records is unavailable, so the stability of deepbite correction, mandibular midline maintenance, and the deliberate functional class II molar finish on the right cannot be determined. No comparator protocol (e.g., 4-premolar extraction, non-extraction distalization, or asymmetric functional appliance therapy) was included, precluding conclusions about relative efficiency. Finally, occlusal function (contact pattern and lateral guidance), periodontal metrics, and retention adherence were not systematically documented, which may influence posttreatment stability and generalizability.

## 4. Conclusions

This case report describes the orthodontic management of a 39-year-old female patient with a skeletal class II pattern, class II division 2 features, deep bite, right side subdivision, and a rightward mandibular midline deviation, treated with an asymmetric 3-premolar extraction protocol (both maxillary first premolars and the mandibular second premolar on the right). Using controlled incisor mechanics and vertical control, we resolved maxillary anterior crowding, corrected the mandibular midline, and normalized overjet and overbite with improved smile esthetics and reduced perceived protrusion. The occlusion was finalized as class I on the left and a deliberate functional class II molar on the right, consistent with the asymmetric objectives. No clinically relevant periodontal complications or root resorption were observed on routine radiographic follow-up, supporting the biological soundness of this compliance-light strategy for carefully selected adult class II subdivision cases with division-2 and deep-bite characteristics.

## Author contributions

**Conceptualization:** Viet Anh Nguyen.

**Data curation:** Viet Anh Nguyen.

**Formal analysis:** Viet Anh Nguyen.

**Funding acquisition:** Viet Anh Nguyen.

**Investigation:** Viet Anh Nguyen, Thi Bao Yen Ngo.

**Methodology:** Viet Anh Nguyen.

**Project administration:** Viet Anh Nguyen.

**Resources:** Viet Anh Nguyen.

**Software:** Viet Anh Nguyen, Thi Bao Yen Ngo.

**Supervision:** Viet Anh Nguyen.

**Validation:** Viet Anh Nguyen.

**Visualization:** Viet Anh Nguyen.

**Writing** – **original draft:** Viet Anh Nguyen, Thi Bao Yen Ngo.

**Writing** – **review & editing:** Viet Anh Nguyen.
